# Surface Modifications of Silver Nanoparticles with Chitosan, Polyethylene Glycol, Polyvinyl Alcohol, and Polyvinylpyrrolidone as Antibacterial Agents against *Staphylococcus aureus*, *Pseudomonas aeruginosa*, and *Salmonella enterica*

**DOI:** 10.3390/polym16131820

**Published:** 2024-06-27

**Authors:** Linh Doan, Quynh N. Le, Khoa Tran, An H. Huynh

**Affiliations:** 1 Department of Chemical Engineering, International University—Vietnam National University Ho Chi Minh City, Ho Chi Minh City 70000, Vietnam; 2 Nanomaterials Engineering Research & Development (NERD) Laboratory, International University—Vietnam National University Ho Chi Minh City, Ho Chi Minh City 70000, Vietnam; tldkhoa@hcmiu.edu.vn; 3 School of Chemical and Environmental Engineering, International University—Vietnam National University Ho Chi Minh City, Ho Chi Minh City 70000, Vietnam

**Keywords:** *S. aureus*, *P. aeruginosa*, *S. enterica*, antimicrobial, composite

## Abstract

In medicine, the occurrence of antibiotic resistance was becoming a critical concern. At the same time, traditional synthesis methods of antibacterial agents often lead to environmental pollution due to the use of toxic chemicals. To address these problems, this study applies the green synthesis method to create a novel composite using a polymer blend (M8) consisting of chitosan (CS), polyethylene glycol (PEG), polyvinyl alcohol (PVA), polyvinylpyrrolidone (PVP), and silver nanoparticles. The results show that the highest ratio of AgNO_3_:M8 was 0.15 g/60 mL, which resulted in a 100% conversion of Ag^+^ to Ag^0^ after 10 h of reaction at 80 °C. Hence, using M8, Ag nanoparticles (AgNPs) were synthesized at the average size of 42.48 ± 10.77 nm. The AgNPs’ composite (M8Ag) was used to inhibit the growth of *Staphylococcus aureus* (*SA*), *Pseudomonas aeruginosa* (*PA*), and *Salmonella enterica* (*SAL*). At 6.25% dilution of M8Ag, the growth of these mentioned bacteria was inhibited. At the same dilution percentage of M8Ag, *PA* was killed.

## 1. Introduction

Microorganisms have a significant impact on human health, particularly bacteria prevalent in numerous surroundings such as food and drinking water, among the most common of which are *Staphylococcus aureus* (*SA*), *Pseudomonas aeruginosa* (*PA*), and *Salmonella enterica* (*SAL*). All of them can cause infections, ranging in intensity and variety, from skin infections to gastrointestinal and respiratory infections [1,2,3].

*Staphylococcus aureus* (*SA*) is a Gram-positive cocci in the *Micrococcaceae* family which develops gleaming, smooth, complete, elevated, and transparent colonies with a golden color and 1 to 4 mm in diameter [4]. *SA* generates tissue-degrading enzymes such as protease, lipase, and hyaluronidase [5]. These bacterial metabolites may help in the spread of infection to nearby tissues. *S. aureus* has a predisposition for spreading to certain organs, including the bones, joints, kidneys, and lungs [6]. In addition, *SA* can cause skin infections (such as boils and cellulitis), respiratory infections, and food poisoning [7]. 

*Pseudomonas aeruginosa* (*PA*) is a Gram-negative, heterotrophic, motile bacteria of the *Pseudomonadaceae* family [8]. *PA* is a rod-shaped bacteria of around 1–5 µm in length and 0.5–1.0 µm in width [8] that forms large, opaque, flat colonies with uneven borders. It is known to cause opportunistic infections, especially in people with weaker immune systems or cystic fibrosis [8]. *P. aeruginosa* has the potential to cause infections in a variety of organs, including the lungs, urinary tract, and skin. On the clinical level, patients with impaired immune systems, including patients with cystic fibrosis, HIV/AIDS, cancer, burn and eye injuries, and non-healing diabetic wounds, are at greatest risk [8].

*Salmonella enterica* (*SAL*), as a group, is a Gram-negative, non-spore-forming prokaryote belonging to the Enterobacteriaceae family [9]. Salmonella range in size from 0.7–1.5 mm to 2.2–5.0 mm, and colonies commonly measure 2–4 mm in diameter [10]. *SAL* infection can cause a systemic illness known as enteric fever, an intestinal infection known as gastroenteritis, or a blood infection in humans known as bacteremia [11].

*SA*, *PA*, and *SAL* growth can be inhibited, or these bacteria can even be killed, using antibiotics [12,13]. However, using antibiotics can cause antibiotic resistance (ABR) [14]. Hence, a material that could inhibit the growth of these bacteria, or even kill these bacteria, without causing ABR phenomena would be ideal. To achieve this, researchers used various antibacterial agents that were not conventional and/or traditional antibiotics such as polymers, metal oxides, or other types of composites. One of the most common antibacterial agents are metal oxides and other metal-type particles such as silver particles [2,3,15,16,17,18], iron oxide nanoparticles [19,20,21], calcium oxide nanoparticles [22], zinc oxide and copper oxide nanoparticles [13,23,24]. However, out of all metal oxides or metal composites, silver particles are the most common antibacterial material. 

Metals like silver are widely employed as active agents to create antibacterial surfaces due to their potent antibacterial properties, even at extremely low doses [25]. Metals, especially silver (Ag), have long been known as efficient antibacterial compounds capable of destroying bacteria [26]. Hence, silver has been utilized for its antibacterial properties even before the pharmaceutical antibiotic revolution. Polymer–metal composites have evolved as a highly effective technique for various surface applications. These silver polymeric materials can also be used in the form of gels or patches for topical applications such as cosmetics or medicine administration and require a balance of physical strength and antibacterial activity [27]. The biological activity of silver particles is influenced by factors such as surface chemistry, size, shape, particle morphology and composition, coating/capping, agglomeration, and dissolution rate. Additionally, particle reactivity in solution, cell type, and the type of reducing agents used for silver particle synthesis play crucial roles in determining their antibacterial efficacy [28].

The methods for synthesizing silver particles, including biological, chemical, and physical approaches, each have their advantages and disadvantages [29]. For instance, the chemical method uses hazardous materials, generates toxic byproducts, requires a lot of energy, and has the potential to harm the environment [28,29]. The physical synthesis process requires sophisticated equipment and procedures, consumes a lot of energy, lacks stabilizing or capping chemicals to avoid agglomeration, and has limits in managing the size and form of silver particles [28,29]. The most significant advantage of silver particles is their environmental friendliness. Green synthesis approaches use reducing biological agents, such as plant extracts or microbial components, to aid in the reduction of silver ions into silver particles. These approaches frequently remove the requirement for external capping and stabilizing agents, minimizing the usage of potentially dangerous compounds throughout the synthesis process [29]. Furthermore, green synthesis methods are seen as more sustainable and ecofriendly than standard chemical synthesis processes, harmonizing with the worldwide effort to achieve sustainable development goals while lowering environmental impacts [29].

Green synthesis and the antimicrobial activities of silver particles involved in it were investigated extensively in materials such as CS/PVA/Ag [30,31], CS/Ag [32,33,34], PVP/CS/Ag [35], PEG/Ag [36,37], PVP/Ag [38], CS/PEG/Ag [39,40,41], and PVA/Ag [12,33,42]. According to Linh et al. [43], a novel polymeric material, M8, which can inhibit the growth of *SA*, consists of PVA, PVP, PEG, and CS. Combining the ability to synthesize silver particles using CS, PVA, PVP, and PEG individually and the anti-*SA* polymer blend M8, in this study, a novel antibacterial agent using silver particles and M8 were synthesized.

## 2. Materials and Methods

### 2.1. Materials

All the chemicals were from the same source as previous study [43]. Additionally, silver nitrate (AgNO_3_, 99.8%) was bought from Shanghai Zhanyun Chemical Co., Ltd. (Shanghai, China). All chemicals were used as received. The School of Biotechnology, International University—Vietnam National University (Ho Chi Minh City, Vietnam) provided *Staphylococcus aureus* strain ATCC 29523 and *Salmonella enterica* ATCC 14028. The Research center for Infectious Disease, International University—Vietnam National University (Ho Chi Minh City, Vietnam) provided *Pseudomonas aeruginosa* strain ATCC 9027.

### 2.2. Methods

#### 2.2.1. Synthesis of M8/Ag Composite

First, the M8 synthesis was based on the publication [43]. Then, 60 mL of M8 was heated to 80 °C. Specifically, the PVA, PVP, and PEG were separately dissolved in distilled water (DI) at a concentration of 0.02 g/mL. CS was mixed with a 3% acetic acid solution at an amount of 0.01 g/mL. To dissolve polymers completely, stirring and heating for a duration of 40 min was carried out. Next, the polymers using a PVA:PVP:PEG:CS:DI ratio of 1:1:1:1:6 (*v*/*v*) were stirred regularly for 1 h to achieve an ideal mixture, referred to as M8. Then, different masses of AgNO_3_ were added into the polymeric mixture as shown in Table 1. 

After adding the silver nitrate, the mixtures were heated and stirred at 80 °C for 6 h. To determine the conversion percentage of Ag^+^ to Ag^0^, every hour, the aliquots were measured using UV–Vis spectrophotometer (JASCO V-730, Tokyo, Japan) at a scanning speed of 40 nm/min and the wavelength range of 400 nm to 500. Then, the sample with the highest conversion was allowed to react for 10 h to determine the reaction time required to reach equilibrium. 

#### 2.2.2. Characterization

The M8Ag and M8 composite were characterized to investigate their chemical and physical properties. Fourier-transform infrared spectroscopy (FTIR, LUMOS, Bruker, Billerica, MA, USA) was utilized to identify the functional groups present in compounds. Subsequently, field emission scanning electron microscopy (FE-SEM, Hitachi SU8000, Tokyo, Japan) was employed to investigate the surface morphology (particle sizes and shapes). Following this, X-ray diffraction spectroscopy (XRD, Bruker D-76187, Karlsruhe, Germany) was employed to analyze the phase composition and crystal structure of the material. Additionally, energy dispersive X-ray spectroscopy (EDX, JEOL JED-2300, Tokyo, Japan) was used to analyze the elemental composition of the composite.

#### 2.2.3. Antibacterial Activity

First, the culture broth and the bacteria were prepared in a manner similar to that described in our previous publication [43]. From each isolate agar plate, a single morphologically comparable colony was chosen and transferred into a glass tube containing 10 mL of Mueller Hinton broth (Himedia, Maharashtra, India) (MHB). The tubes were incubated at 35 °C for 24 h. Subsequently, optical density at a wavelength of 600 nm (OD600) was measured using Hach DR6000 spectrophotometer (Hach, Loveland, CO, USA), and the bacterial suspension was diluted to achieve an OD600 of 0.01 [44]. This process was repeated for three types of bacteria: *SA*, *PA*, and *SAL*. Then, to determine the minimum inhibition concentration (MIC) and the bactericidal effects, the MIC experiment was performed in a manner similar to that described in the previous publication, without any modifications [43]. After obtaining the inhibition percentage, the three best-performing antibiotic concentrations (M8Ag and M8) were chosen and spread evenly onto separate Mueller Hinton agar plates. Subsequently, the plates were incubated at 35 °C for 24 h, and then we identified the presence of bacteria colonies on each dish.

## 3. Results and Discussion

### 3.1. Determining the Conversion Percentage of Ag^+^ to Ag^0^ from AgNO_3_ and M8

**Figure 1:**
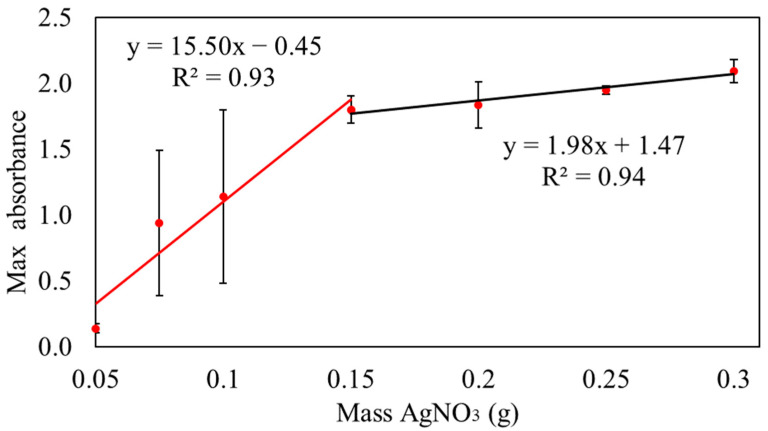
Absorbance of samples after 6 h at wavelength of 455 nm.

Measuring the absorbance at 455 nm, samples with AgNO_3_ masses ranging from 0.05 to 0.15 g continued to exhibit ongoing reactions after 6 h, achieving a 100% conversion rate, as shown in Figure 1.

The mechanism of converting Ag^+^ to Ag^0^ in the M8 polymer blend can be explained as the electron was exchanged. M8 availability was limited, and the increases in the concentrations of AgNO_3_ caused an increase in Ag^+^ concentration. Insufficient M8 led to the reduction of Ag^+^ to Ag^0^ conversion, which accounted for the observed slope variations and the appearance of two different trends in the graph [45]. The conversion percentage of Ag^+^ to Ag^0^ in sample S1 was excessively low, while samples S2 and S3 exhibited large error bars, rendering the data unreliable. Conversely, samples S5, S6, and S7 were deemed unsuitable for further analysis as they were nearing equilibrium, which produces less Ag^+^ than S4. Hence, sample S4 was determined to be the maximum mass of silver nitrate to obtain 100% conversion of Ag^+^ to Ag^0^. However, to determine the equilibrium time to produce Ag^0^, sample S4 reacted for 10 h.

**Figure 2:**
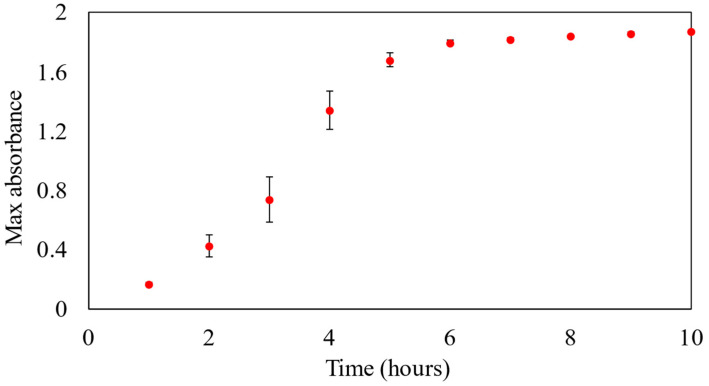
Absorbance of samples for 10 h at wavelength of 455 nm.

Figure 2 shows the absorbance of the M8/AgNO_3_ (S4 sample) solution for 10 h, with absorbance measurements taken at hourly intervals.

As shown in Figure 2, initially, within the first 6 h of the reaction, a rapid increase in absorbance values was observed. This indicated that the reaction between M8 and AgNO_3_ was proceeding vigorously and with high efficiency during this initial phase. However, after the first 6 h, the rate of growth in absorbance values began to slow down, gradually stabilizing in the subsequent hours. This might suggest the approach of the reaction to the equilibrium state at 10 h. Based on the previous literature, all the components of M8 can reduce Ag^+^ to Ag^0^ [12,30,31,32,33,34,35,36,37,38,39,40,41,42]. However, to determine which component can reduce the silver ions the most, the reduction mechanisms, and the synergistic effects between these polymers in the reduction process, further investigation should be conducted in the future. 

**Figure 3:**
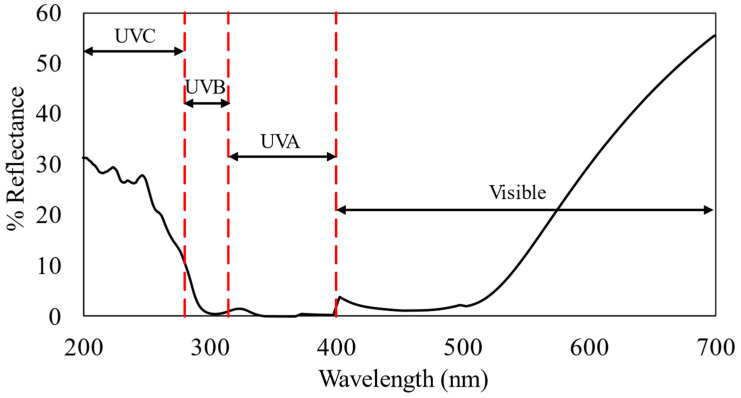
Reflectance percentage (200–700 nm) of M8Ag after reacting for 10 h.

Afterward, the reflectance percentage of sample S4 from 200 to 700 nm was investigated, as shown in Figure 3.

As illustrated in Figure 3, the S3 composite displayed reflectance in the UV-C range (below 280 nm) ranging from 11% to 31%, below the 11% reflectance in the UV-B range (280–315 nm), and less than the 2% reflectance in the UV-A range (315–400 nm). Reflectance in the visible light spectrum ranged from 2% to 65%. These findings suggested that the material demonstrates moderate reflectivity in visible light.

### 3.2. Characterization

#### 3.2.1. FE-SEM

**Figure 4:**
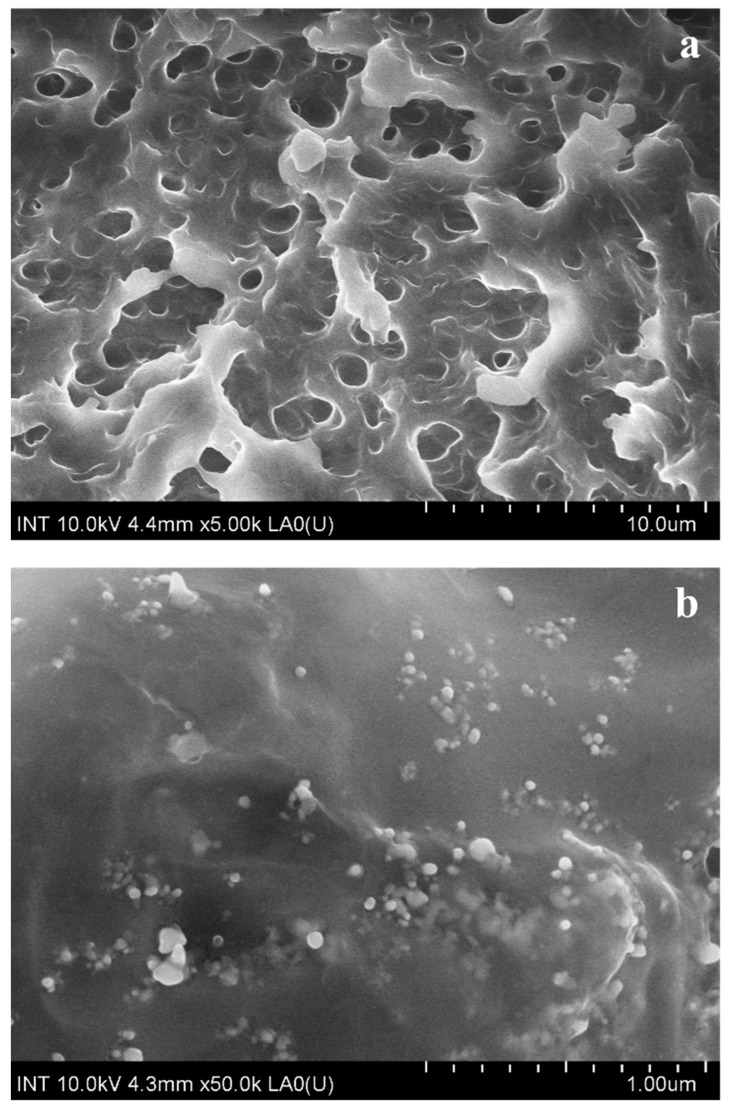
FE-SEM image of (**a**) M8 and (**b**) M8Ag.

Using FE-SEM, the morphology of M8 and M8Ag were determined, as shown in Figure 4.

**Figure 5:**
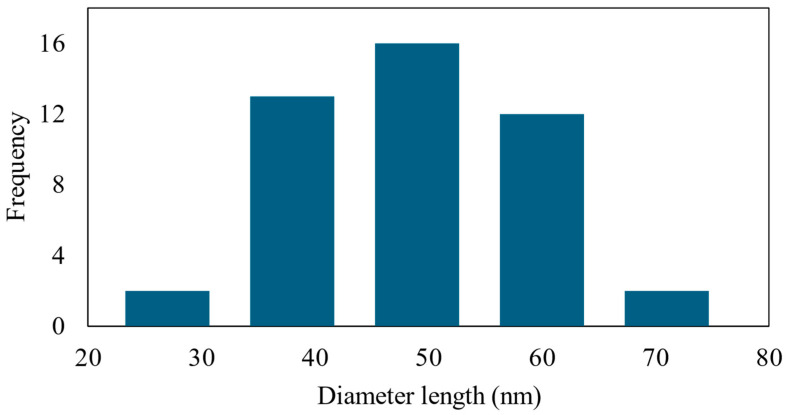
Size distribution of AgNPs in M8Ag composite.

As shown in Figure 4a, M8 exhibited pores without any discernible small dots. In contrast, M8Ag (Figure 4b) displayed small round dots, indicating the presence of these particles. Using ImageJ software (version 1.53e) and the FE-SEM images shown in Figure 4b, the size and size distribution of the silver particles were determined, as shown in Figure 5. 

As shown in Figure 5, the size distribution of particles in M8Ag sample was determined to range from 35 nm to 50 nm, with the highest frequency observed in the 40–45 nm size range. Additionally, there was a noticeable trend towards larger sizes, indicating the presence of a small number of particles exceeding 50 nm. Hence, the average size of the AgNPs was 42.48 ± 10.77 nm. However, to confirm whether these nanoparticles were silver necessitated the use of the XRD method.

#### 3.2.2. XRD

**Figure 6:**
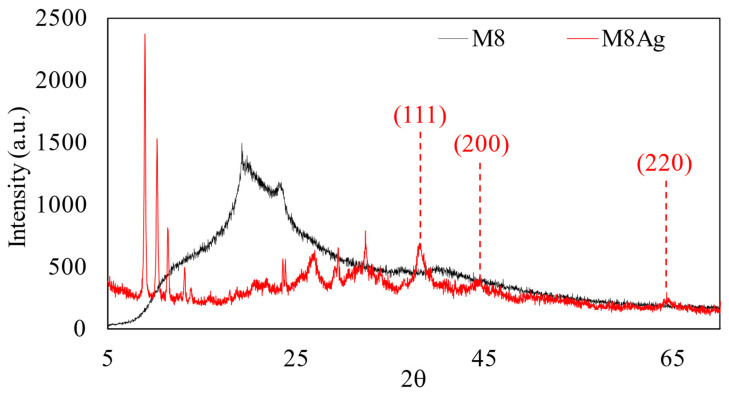
XRD analysis of M8 and M8Ag composite.

XRD was used to analyze the phase composition and crystal structure of M8 and M8Ag. The XRD analysis is shown in Figure 6. 

As shown in Figure 6, the peaks at 2θ values of 38°, 44°, and 65° correspond to the *hkl* indices of (111), (200), and (220), indicating spherical and crystalline Ag particles [32,46]. Using the Scherrer equation (the shape factor and X-ray wavelength are 0.94 and 1.5406 Å, respectively) [47], the determined average crystallite size of Ag was 44.53 nm, consistent with the results obtained from FE-SEM measurements (42.48 ± 10.77 nm). Hence, using XRD and FE-SEM, silver nanoparticles (AgNPs) were presented in M8–polymer blends consisting of CS, PVA, PEG, and PVP. To be certain, EDX was used once again to identify whether the particles were constituted of Ag and to calculate their percentage inside M8. 

#### 3.2.3. EDX

EDX was used to analyze the elemental composition of M8 and M8Ag, as shown in Table 2.

In Table 2, it can be seen that Ag provided 39.22 ± 0.34% of the mass in M8Ag. This suggests that the remainder of the matrix was composed of polymers (PEG, PVP, PVA, and CS). Additionally, M8 exhibits a minimal presence of sodium (Na), silicon (Si), calcium (Ca), and iron (Fe), all below 1%, implying the impurities of the raw materials, as well as the synthesis process, which contributed negligibly to the material’s composition. Conversely, M8Ag reveals the presence of chlorine (Cl) at 0.15 ± 0.02% and potassium (K) at 3.20 ± 0.07%, elements absent in M8. However, during the AgNPs’ synthesis, no chemicals that contain Cl and K were used. Hence, these elements can be considered as contamination during the synthesis. Additionally, the atom fraction of Ag in M8Ag was determined, as shown in Table 3. 

As shown in Table 2 and Table 3, and combined with the data from FE-SEM and XRD, 39.22 ± 0.34% (mass fraction) and 8.1 ± 0.07% (atomic fraction) of silver nanoparticles (AgNPs) at the size of 42.48 ± 10.77 nm (FE-SEM) or 44.53 nm (XRD) were presented in M8–polymer blends consisting of CS, PVA, PEG, and PVP. 

#### 3.2.4. FTIR

**Figure 7:**
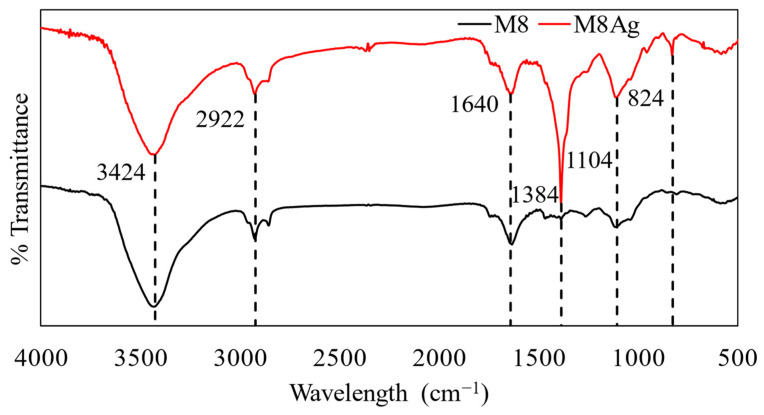
FTIR spectra of M8 and M8Ag.

FTIR was utilized to identify the functional groups present in compounds (Figure 7).

For M8, the peak at 3424 cm^−1^ may correspond to the stretching vibration of -OH groups with primary -NH groups of CS [43,48]. The peak observed at 1640 cm^−1^ could be attributed to various functional groups, including the C=O stretching vibration of PVP, the bending mode of O-H groups (due to water presence), the C=O stretching vibration of PEG, or the C=O stretching (Amide I) of CS + AA [43,48]. Additionally, the peak at 1104 cm^−1^ might correspond to the stretching vibrations of C-O bonds, the symmetric stretching of C-O-C in PEG, or the shift of the free amino group (-NH_2_) at the C2 position of glucosamine in CS + AA [43,48].

Since M8Ag was synthesized based on M8, it shares similarities in the functional groups present in compounds. In contrast, the FTIR spectra of the silver nanoparticles in the M8Ag sample revealed prominent peaks at 2922 cm^−1^, 1640 cm^−1^, and 1384 cm^−1^. A sharp and intense absorption band at 1640 cm^−1^ was attributed to the stretching vibration of the (NH) C=O group. As mentioned, another much sharper and more intense peak at 1384 cm^−1^ in the M8Ag compared to M8 indicated the C-C and C-N stretching. Furthermore, the presence of a sharp peak at 2922 cm^−1^ was assigned to the stretching vibration of C-H and C-H (methoxy compounds). Additionally, in the M8Ag sample, a new distinct peak was found at 824 cm^−1^. Due to the similarities, it is safe to conclude that in the M8Ag sample, M8 still exists, and some changes in the bonds between the polymers in M8 and the silver particles led to some shifts, increases, and changes in the peaks. This indicated the M8Ag was successfully synthesized.

### 3.3. Antibacterial Activity

**Figure 8:**
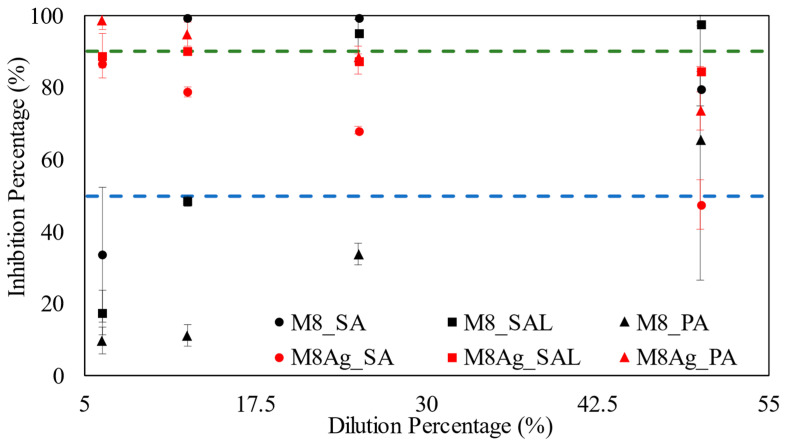
MIC results: the blue line represents MIC_50_, green line represents MIC_90_. Triangle, square, and round dots represent *PA*, *SAL*, and *SA*, respectively. Red color and black color represent M8Ag and M8, respectively.

Figure 8 presents the minimum inhibitory concentration (MIC) results for three types of bacteria: *SA*, *SAL*, and *PA* from 5% to 50% dilution. 

For *SA* bacteria, M8Ag had an MIC_50_ value of 6.25%. Comparing to M8 from previous publication, M8Ag has a smaller MIC_50_ value [43]. However, M8Ag did not display any inhibitory activity at MIC_90_, regardless of the dilution percentage. Comparing to the M8 from previous publication, M8Ag has inferior inhibition ability [43]. In the case of *SAL* bacteria, M8Ag exhibited an MIC_50_ value of 6.25% and an MIC_90_ value of 12.5%, indicating its efficacy against *SAL* bacteria. However, against *SAL*, M8 has the MIC_90_ at 25% dilution. This indicates that M8Ag has lower MIC_90_ than M8. For *PA* bacteria, M8Ag demonstrated MIC_50_ and MIC_90_ values of 6.25%. On the other hand, against *PA*, M8 does not have MIC90, indicating that M8Ag is superior to M8. Hence, overall, M8Ag is a better antibacterial agent than M8. Notably, from Figure 8, Table 4 focuses on MIC_50_ and MIC_90_ results, referring to the minimum concentrations at which 50% and 90% inhibition of bacterial growth were achieved for M8Ag, respectively.

Table 4 clearly indicates that M8Ag exhibited superior antimicrobial activity against *SAL* and *PA* compared to *SA*, due to its lower MIC_90_ values for inhibiting bacterial growth. 

Using MIC results, the aliquots at concentrations of 12.5% and 6.25% dilution were spread on agar plates to assess the bactericidal abilities of M8Ag. The findings revealed that neither dilution concentration could effectively kill *SA* and *SE* bacteria, as shown in Table 5.

As shown in Table 5, 6.25% M8Ag demonstrated the ability to eradicate *PA* bacteria. Based on both MIC and the bactericidal effect results, 6.25% M8Ag exhibited a strong capability to eliminate *PA*, indicating that *PA* was the most susceptible bacteria to M8Ag. For *SA* and *SAL*, M8Ag only inhibited their growth and did not completely eradicate them.

M8 included PVA, PVP, PEG, and CS. CS and PVA were crucial in their ability to fight against a variety of bacteria [33,43]. However, PVA alone had poor stability in water properties; to overcome this limitation, PVA should be mixed with PVP, which covers the surface particles to produce a stable colloid [43]. The combination of PEG and PVA produced strong interactions via hydrogen bond formation, which improved the mixture’s overall stability and properties [43]. Meanwhile, the PVA/PEG blend decreased adhesion to bacterial surfaces, which added to the particles' antibacterial abilities [49,50]. Specifically, the hydrophilicity of PVA worked as a regulating element, lowering protein adsorption and cell adhesion [49]. Hence, PVA appeared to operate as a barrier against bacterial adherence on the catheter surface [49]. In addition, PEG worked as a reducing agent, speeding up the reduction of silver ions to silver particles and allowing for the efficient production of silver particles with improved antibacterial activity [40].

On the other hand, silver particles released silver ions continuously, acting as a mechanism of antibacterial activity [51]. These ions adhered to cell walls and membranes due to electrostatic attraction and sulfur protein affinity [52]. This adhesion enhanced membrane permeability, disrupting the bacterial envelope. Inside cells, silver ions deactivated respiratory enzymes, generating reactive oxygen species, which halted adenosine triphosphate (ATP) production and caused deoxyribonucleic acid (DNA) modification [51]. The interaction between silver ions and the sulfur and phosphorus components of DNA can disrupt DNA replication and cell reproduction and potentially lead to microbial termination, given the significance of sulfur and phosphorus in DNA’s structure [51]. 

M8Ag combined the advantages of M8 with Ag particles, considerably increasing its antibacterial efficiency. More specifically, the presence of M8 promoted the conversion of Ag^+^ to Ag^0^. In contrast, in an acidic environment (3% acetic acid is used to produce chitosan), bacteria may hydrolyze nearby ester segments and create Ag particles, resulting in enhanced Ag production [52]. As a result, the antibacterial efficiency increases in proportion to the number of Ag particles [51]. 

Gram-negative bacteria have thinner cellular walls than Gram-positive ones, and the strong cellular wall may limit nanoparticle penetration into cells [53]. Hence, M8Ag was more effective against Gram-negative bacteria. However, while both *SAL* and *PA* are Gram-negative bacteria, the antibacterial efficacy of M8Ag against *SAL* is not as strong as against *PA*, which was shown in MIC_90_ and bactericidal effect results. Hence, the antibacterial mechanisms, interaction between the antimicrobial agents and the bacteria, and further investigation for other possible explanations for the aforementioned phenomenon should be the subject of future studies and research. Additionally, researchers should consider studying the cell cytotoxicity and toxicity on mice to provide much deeper understanding of M8Ag.

## 4. Conclusions

The combination of M8 (including PVA, PVP, PEG, and CS) and silver nitrate created a novel composite (M8Ag) that was environmentally friendly, possessed antibacterial properties, and addressed the issue of antibiotic resistance. M8Ag, with an AgNO_3_:M8 ratio of 0.15 g/60 mL, achieved 100% conversion of Ag^+^ to Ag^0^ after reacting at 80 °C for 10 h. However, the reduction mechanism, as well as determining the main reducing agent(s) in M8, should be investigated in the future.

Silver nanoparticles were successfully synthesized using M8 polymer blends, with a mass fraction of 39.22 ± 0.34% and an atomic fraction of 8.1 ± 0.07. The nanoparticles had a size of 42.48 ± 10.77 nm according to FE-SEM analysis and 44.53 nm based on XRD measurements.

The antibacterial efficacy of M8Ag at diluted concentrations varied across bacterial strains. Specifically, for *SA*, a concentration of 6.25% was effective at MIC_50_. *SAL* exhibited susceptibility at both MIC_50_ and MIC_90_, with concentrations of 6.25% and 12.5%, respectively. *PA*, on the other hand, demonstrated sensitivity at MIC_90_, with a concentration of 6.25%.

In conclusion, at 6.25% M8Ag, this dilute concentration served as the MIC_90_ for *PA*, while for the other two strains, it approached the MIC_50_ threshold. This suggested that M8Ag exhibited the strongest antibacterial activity against *PA*. To elucidate further, when spread on agar dishes, 6.25% M8Ag can eliminate *PA* but not *SA* and *SAL*. To elucidate the principle and assess the antibacterial potential of M8Ag against both Gram-negative and Gram-positive bacteria, further investigation into other potential explanations for this observed phenomenon could be pursued as part of future studies. Additionally, the cells’ cytotoxicity and toxicity on mice should be investigated in the future.

## Figures and Tables

**Table 1:** Mass of AgNO_3_ in M8.

Samples	M8 (mL)	AgNO_3_ (g)
S1	60	0.05
S2	60	0.075
S3	60	0.1
S4	60	0.15
S5	60	0.2
S6	60	0.25
S7	60	0.3

**Table 2:** Mass percentage in M8 and M8Ag.

Element	M8 (%)	M8Ag (%)
C	55.51 ± 0.22	18.12 ± 0.11
N	4.36 ± 0.27	9.10 ± 0.25
O	39.05 ± 0.44	30.20 ± 0.38
Na	0.23 ± 0.02	-
Si	0.53 ± 0.03	-
Ca	0.10 ± 0.02	-
Fe	0.23 ± 0.05	-
Cl	-	0.15 ± 0.02
K	-	3.20 ± 0.07
Ag	-	39.22 ± 0.34
Total	100	100

**Table 3:** Atom percentage in M8 and M8Ag.

Element	M8 (%)	M8Ag (%)
C	62.38 ± 0.24	33.57 ± 0.21
N	4.2 ± 0.26	14.44 ± 0.4
O	32.94 ± 0.37	42 ± 0.53
Na	0.13 ± 0.01	-
Si	0.26 ± 0.02	-
Ca	0.03 ± 0.01	-
Fe	0.06 ± 0.01	-
Cl	-	0.1 ± 0.01
K	-	1.79 ± 0.04
Ag	-	8.1 ± 0.07
Total	100	100

**Table 4:** MIC results of M8Ag.

Bacteria	MIC_50_	MIC_90_
*SA*	6.25%	-
*SAL*	6.25%	12.5%
*PA*	-	6.25%

**Table 5:** Bactericidal effects of M8Ag (−) and (+) means negative and positive bacteria growth.

Bacteria	12.50%	6.25%
*SA*	−++	+++
*SAL*	+++	+++
*PA*	+++	−−−

## Data Availability

All data that support the findings of this study are included within the article.
